# Supplementary figures and images for: Resolution of herpes simplex virus reactivation in vivo results in neuronal destruction
Source: PLoS Pathog. 2020 Mar 5;16(3):e1008296. doi: 10.1371/journal.ppat.1008296 (PMC7058292; doi:10.1371/journal.ppat.1008296)

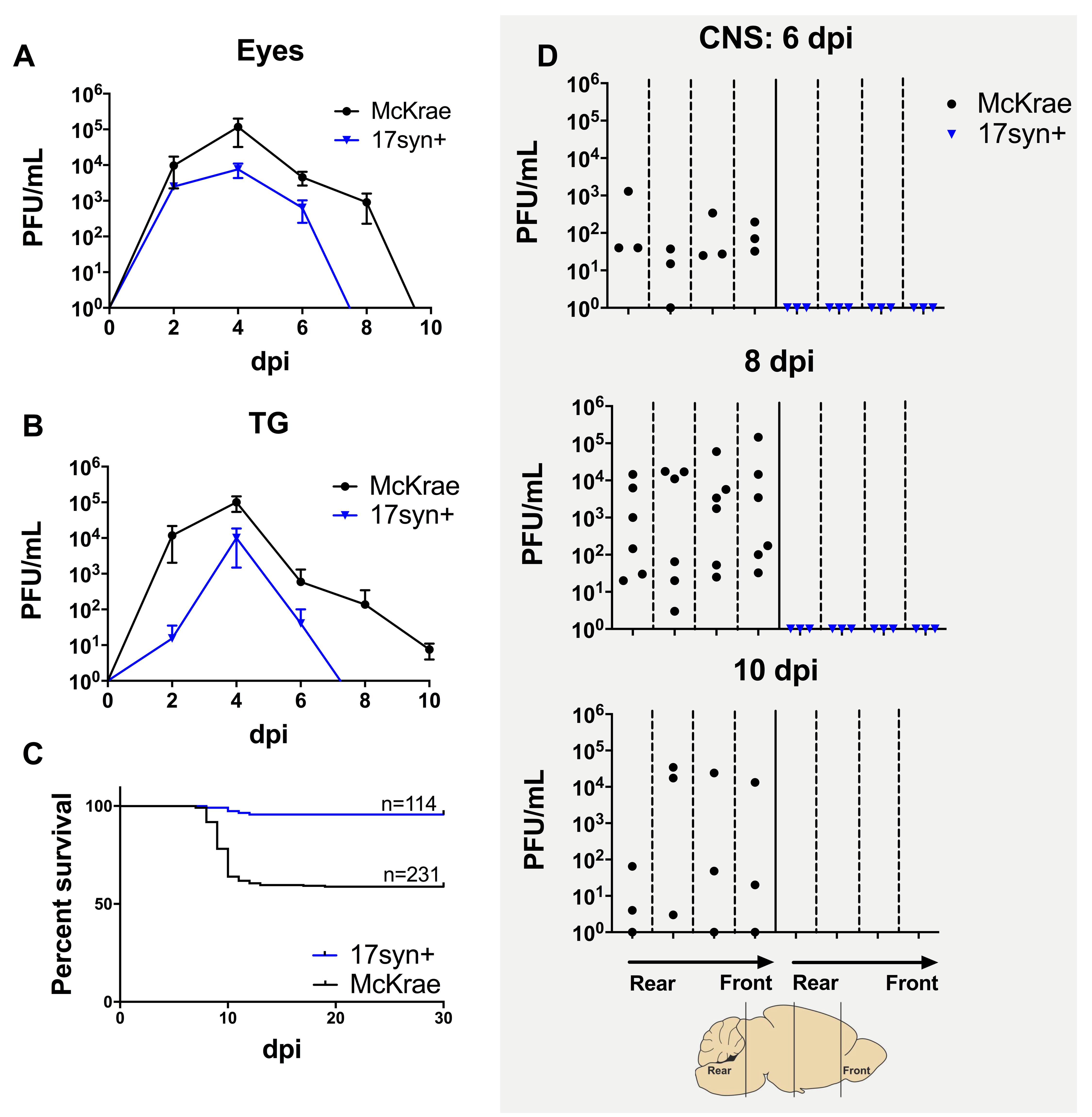

Supplement: S1 Fig — Mice were infected on scarified corneas with 1 x10^5^ PFU of McKrae or 1 x10^6^ PFU of 17syn+ per eye and at the indicated time, tissues from three or more mice were harvested and analyzed for infectious virus titers. (A) Viral replication in the eyes. Infectious virus titers on day 4 post-infection were compared by Student’s t-test; p = 0.0897. (B) Viral replication in the trigeminal ganglia (TG). Infectious virus titers on day 4 post-infection were compared by Student’s t-test; p = 0.0292. (C) Survival curve following infection of C57BL/6 female mice with McKrae or C57BL/6 male mice with 17syn+. Log-rank (Mantel-Cox) p<0.0001. (D) Viral replication in the central nervous system (CNS). High levels of infectious virus were recovered in the CNS of mice with signs of encephalitis (hunching, moribund) while no or low levels of virus were recovered from mice lacking such signs. (TIF) [file ppat.1008296.s001.tif]

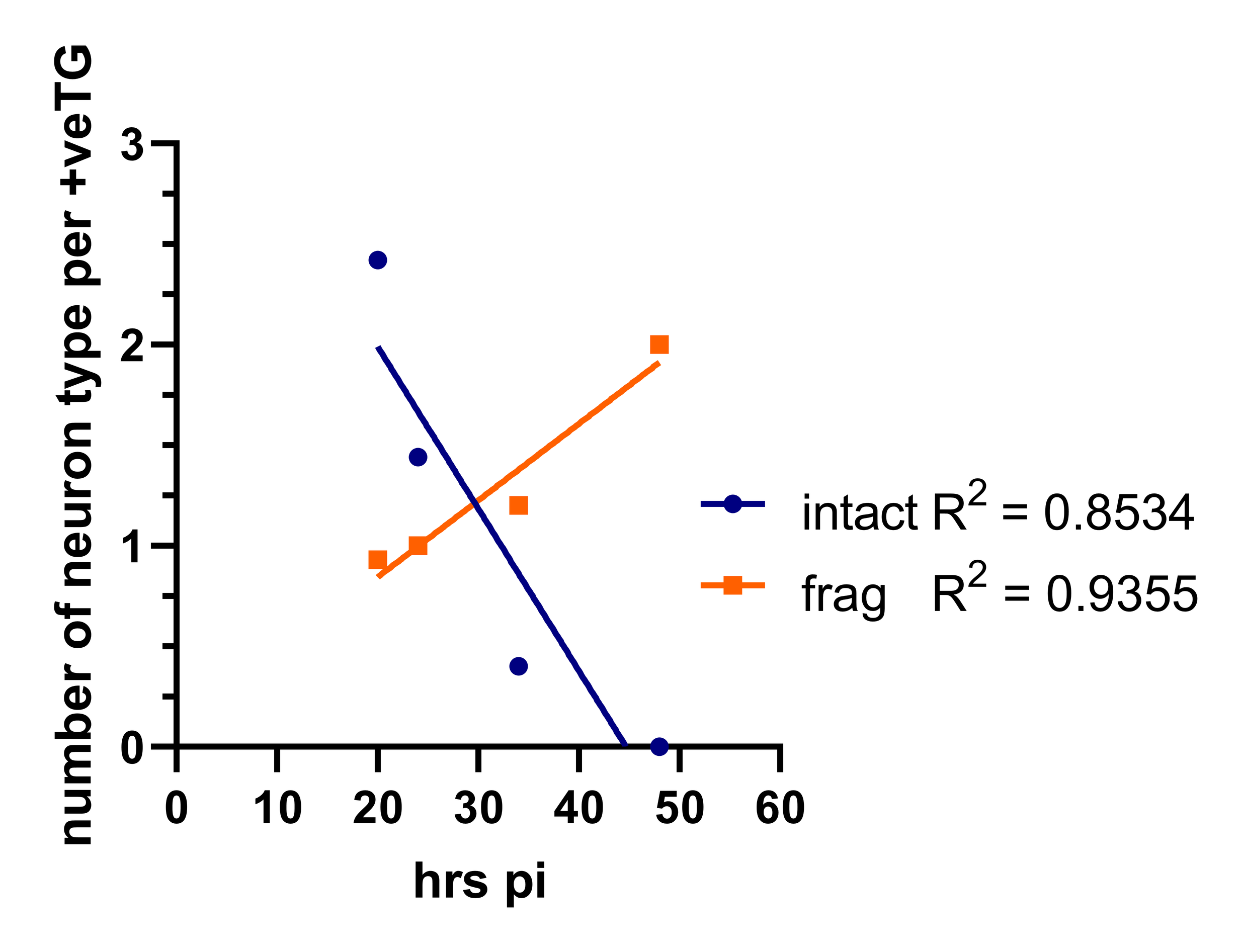

Supplement: S2 Fig — The average number of intact and fragmented neurons in ganglia that contained positive neurons (+veTG) as determined in Fig 1 (minimum of 10 ganglia per time point) was plotted at each time point post-reactivation induction (hrs pi). A negative linear regression was determined for neurons of the intact phenotype and a positive linear regression was found for neurons of the fragmented phenotype. R2 values are given on the graph for each relationship. (TIF) [file ppat.1008296.s002.tif]

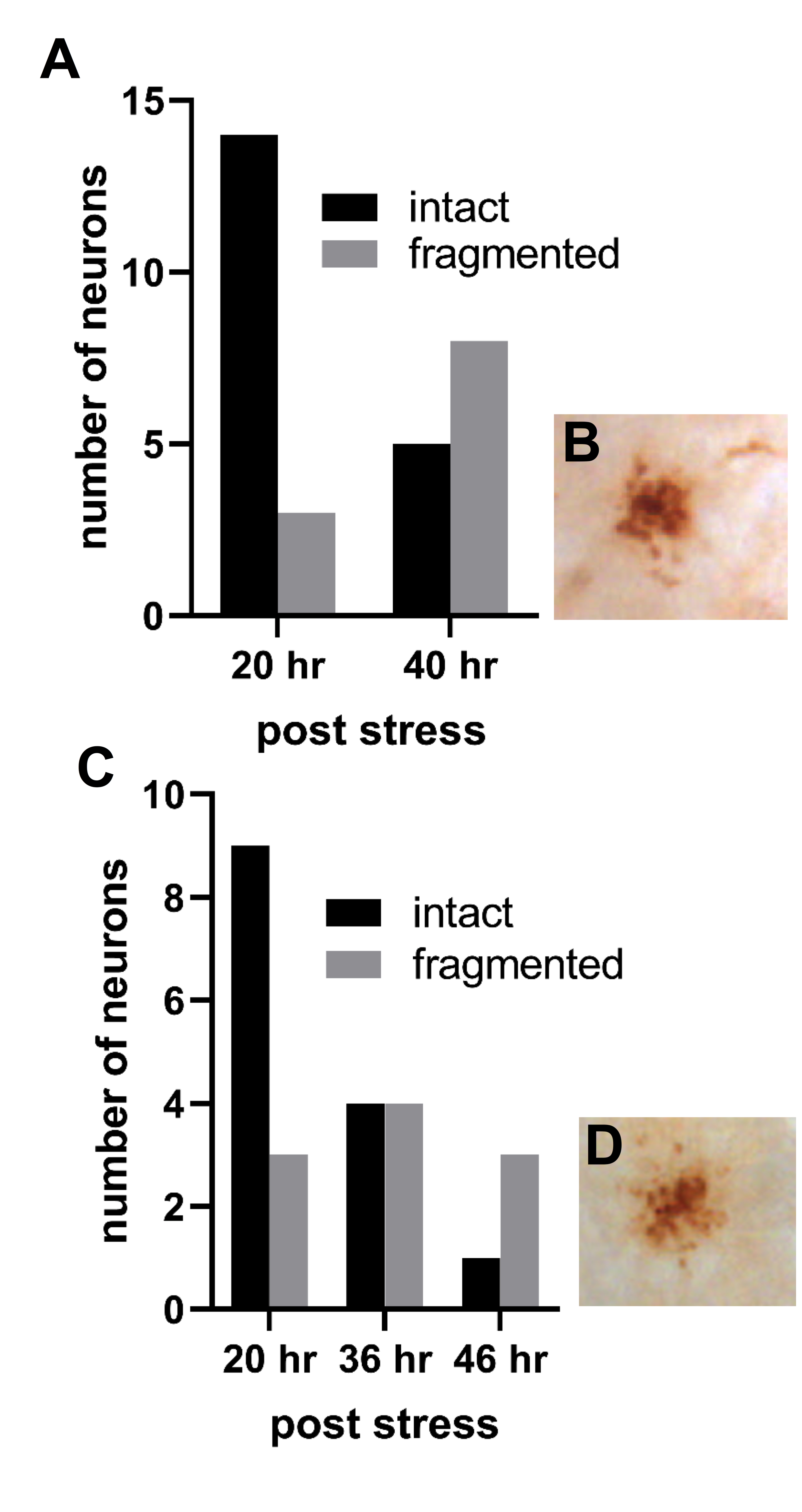

Supplement: S3 Fig — The number of intact and fragmented neurons and examples of individual fragmented neurons in trigeminal ganglia of Swiss Webster mice following reactivation induced by hyperthermic stress (A,B) or in mice following reactivation induced by corneal scarification (C,D) are shown. Bars in A and C indicate the number of intact or fragmented viral protein positive neurons in 10 ganglia/group at each time post-induction. Examples of HSV viral protein positive fragmented neurons are shown in B and D. (TIF) [file ppat.1008296.s003.tif]

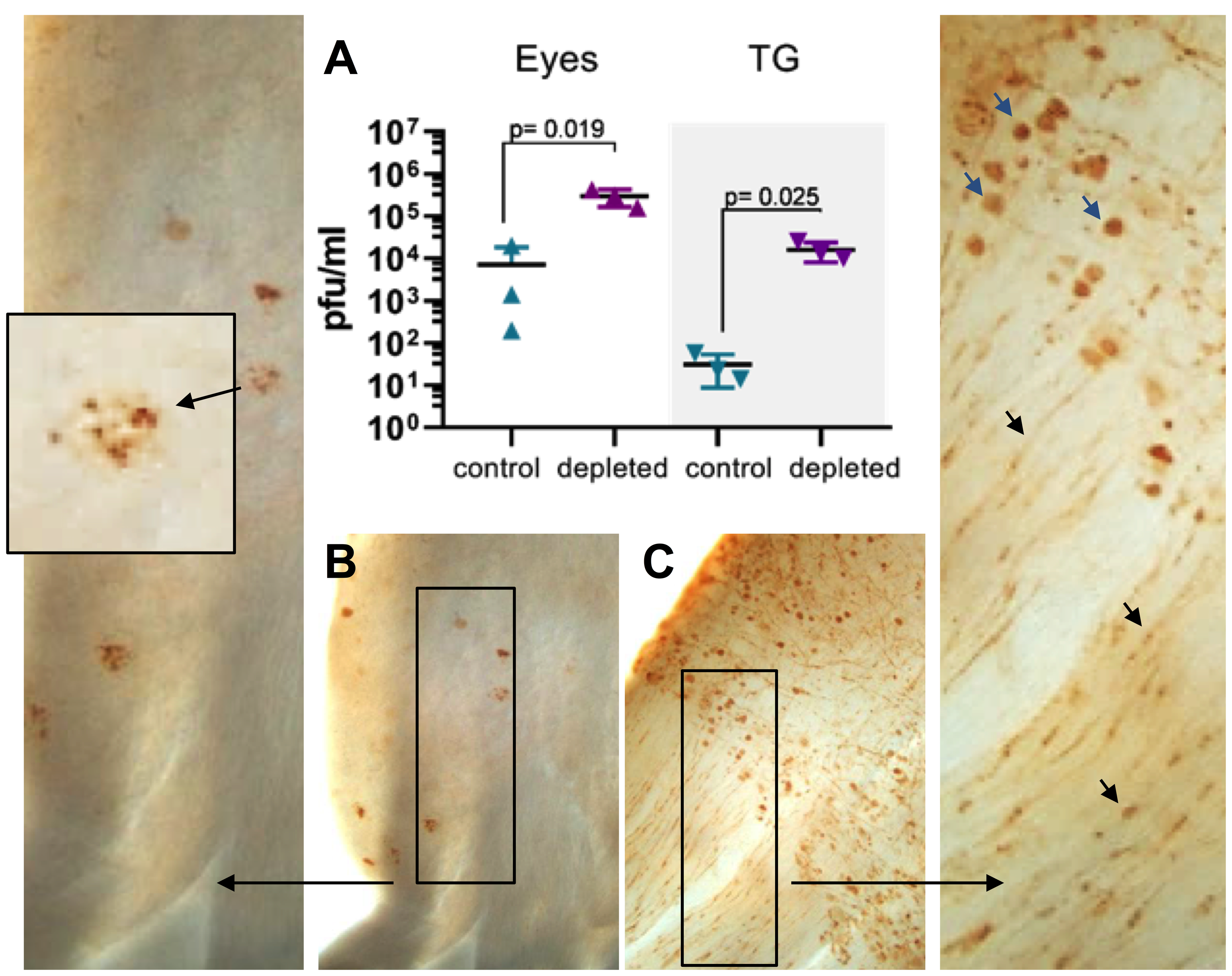

Supplement: S4 Fig — Mice were infected on scarified corneas with 1 x10^6^ PFU of 17syn+ per eye and 1 day later treated with anti- CD4/CD8 depleting/ neutralizing antibodies or control IgG. On day 7 pi, tissues from 3 mice in each group were harvested and analyzed for infectious virus titers or viral protein expression. (A) Viral titers in the eyes and TG from control and anti-CD4/CD8 treated mice. Anti-CD4/CD8 treatment resulted in significantly higher viral titers in both the eyes (Student’s t-test; p = 0.019) and TG (Student’s t-test; p = 0.025). Line indicates average of 3 samples. (B and C) Viral protein expression (brown DAB reaction product) in ganglia from control (B) and anti-CD4/CD8 (C) treated mice. Blue arrows indicate neurons expressing viral proteins. Black arrows indicate viral protein expression in cells lining the axonal tracts. Low power and high power views are shown to emphasize the striking differences in number of infected neurons in TG from anti-CD4/CD8 treated mice. A fragmented neuron is shown at higher power (boxed inset). (TIF) [file ppat.1008296.s004.tif]

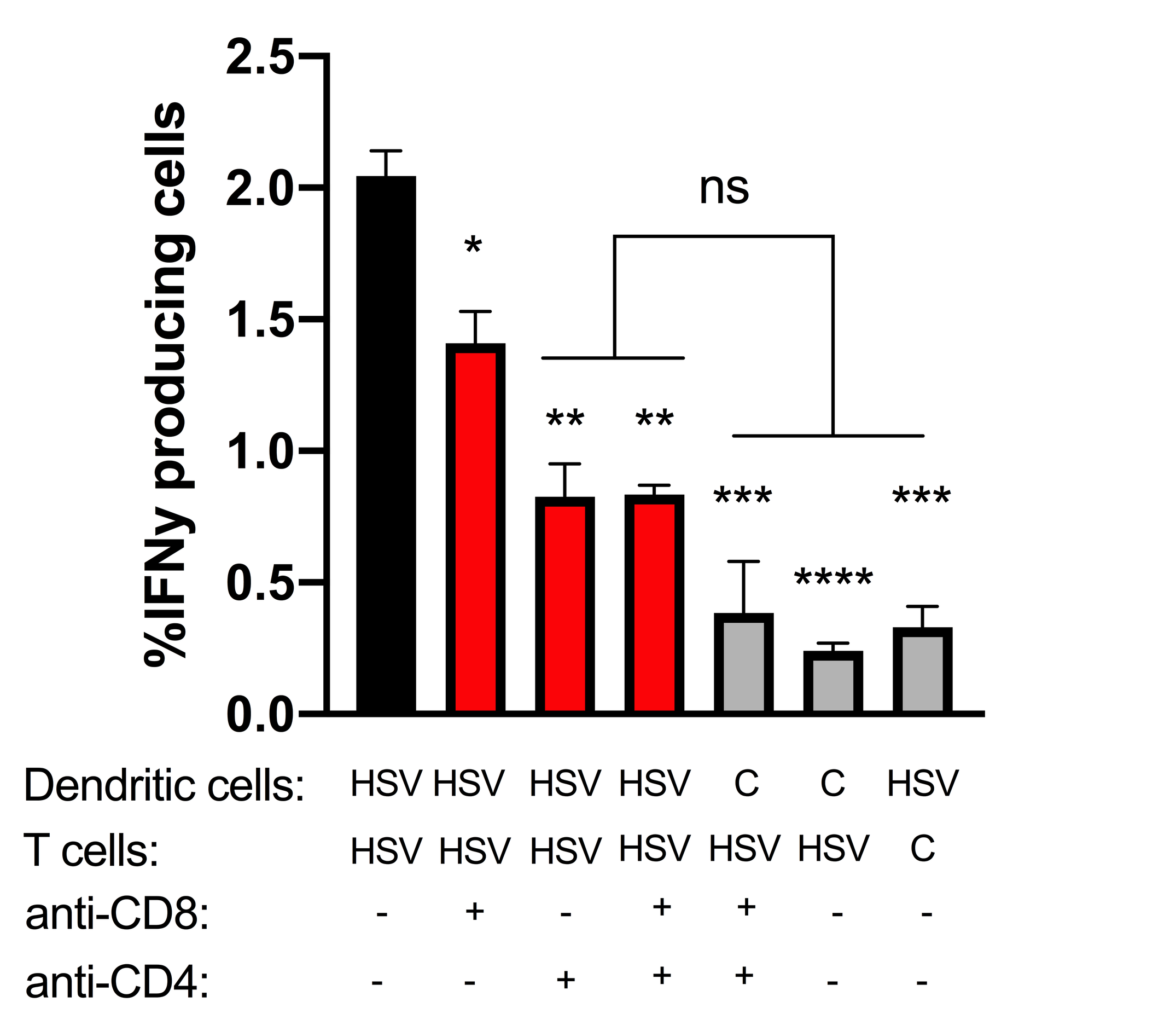

Supplement: S5 Fig — Control T cells (C-T cells) and T cells from HSV infected mice (HSV-T cells) were harvested and incubated with anti-CD4, anti-CD8, or control IgG and presented with HSV1 antigen from dendritic cells (HSV) or unexposed control dendritic cells (C) as detailed in Methods. Production of intracellular IFNγ was measured 70 h later. Addition of both anti-CD4 and anti-CD8 significantly reduced T cell IFNγ production compared to control IgG treated T cells from HSV infected mice presented with HSV antigen exposed dendritic cells. The percent cells producing IFNγ in cultures treated with anti-CD4/CD8 was not significantly different from background levels detected in control conditions. One-way ANOVA with Tukey’s multiple comparison test: * = p<0.05; ** = p<0.01; *** = p<0.001; **** = p<0.0001. (TIF) [file ppat.1008296.s005.tif]

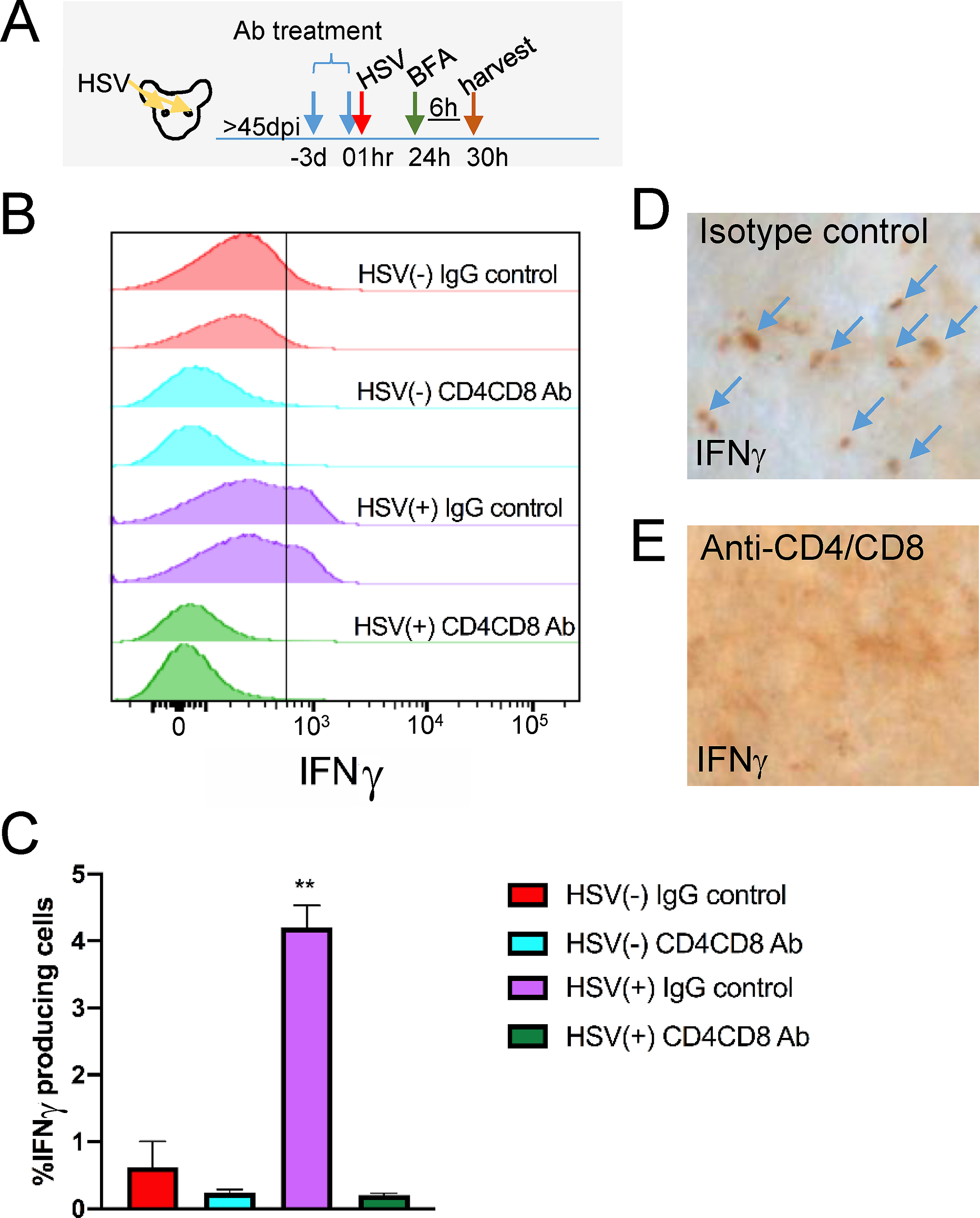

Supplement: S6 Fig — Naïve mice [HSV(-)] and mice latently infected with HSV-1 strain 17syn+ [HSV(+)] were treated with control IgG or anti-CD4 and anti-CD8 depleting antibodies. Mice were given a second dose of antibodies 3 days later and the latently infected mice were re-infected with 1 x10^6^ PFU of 17syn+. Brefeldin A (BFA) was given i.p. 24 post-infection and tissues were harvested 6 hours post-injection. Values from naïve mice are plotted in parallel to determine background fluorescent levels. (A) Schematic representation of experimental approach. (B) Flow cytometric analysis of IFNγ expression in the CD3+ TCRβ+ population. (C) Percent of IFNγ producing cells in the CD3+ TCRβ+ population as determined by flow cytometric analysis. One-way ANOVA with Tukey’s multiple comparison test; ** = p<0.01. (D) Intracellular IFNγ was detected in whole ganglia (brown; DAB reaction product). Arrows indicate cells positive for IFNγ. (TIF) [file ppat.1008296.s006.tif]

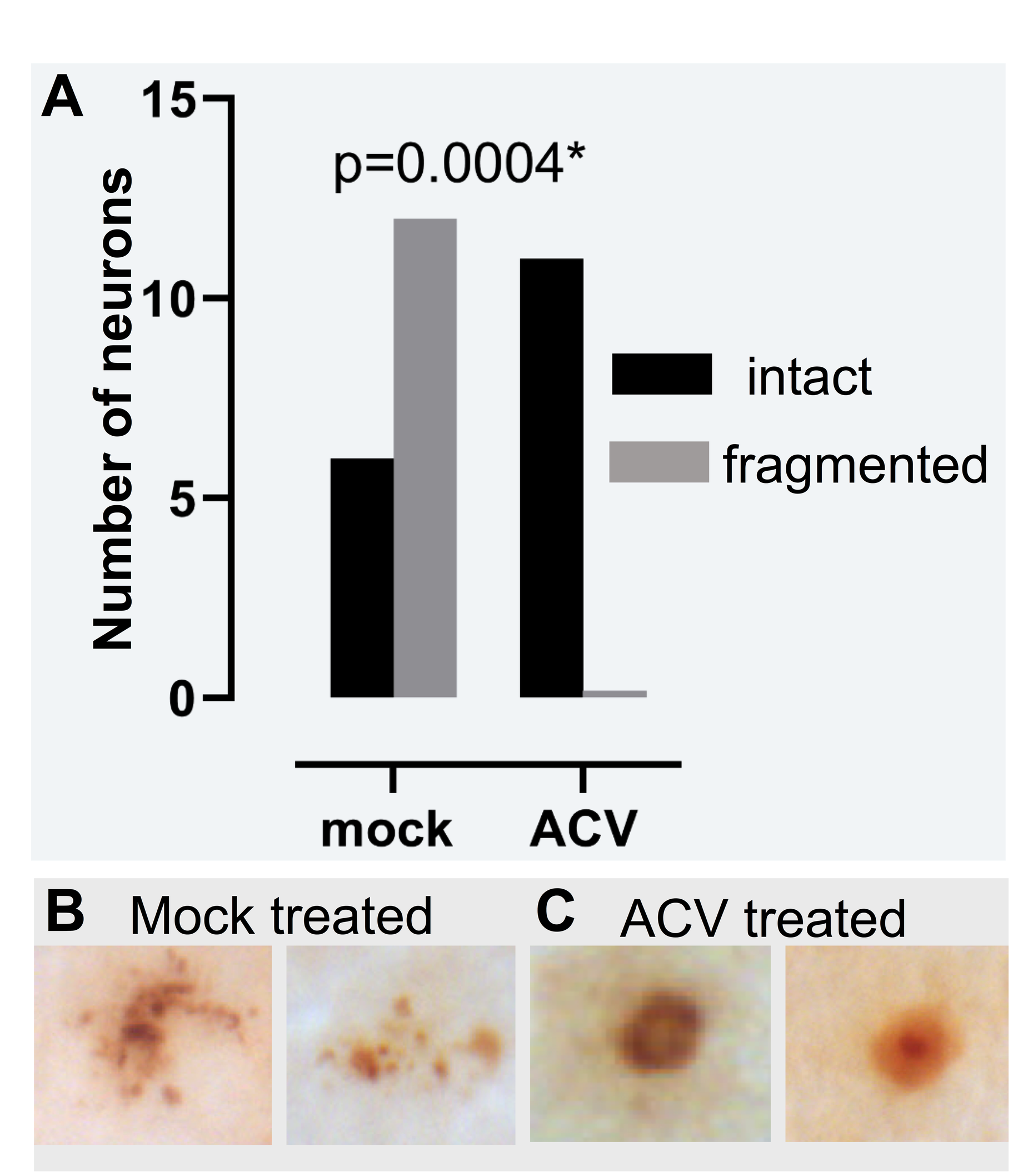

Supplement: S7 Fig — Mice latently infected with 17syn+ were treated with ACV (50 mg/kg, ip, 2x daily) or saline (mock) control (n = 5 mice/group) starting one day prior to hyperthermic stress and continued until the time of sacrifice. At 46 h phs, ganglia were removed and HSV proteins detected by WGIHC. (A) Bars represent the number of intact and fragmented viral protein expressing neurons (n = 10 TG per group). Fragmented neurons were not detected in the ACV treated group. *ACV treated and untreated groups are different, p = 0.0004, Fishers exact test. (B) Photomicrographs of fragmented, HSV protein positive neurons detected in mock treated mice. (C) Photomicrographs of intact, HSV protein positive neurons detected in ACV treated mice. (TIF) [file ppat.1008296.s007.tif]
