# Supplementary material for: Resolution of herpes simplex virus reactivation in vivo results in neuronal destruction
Source: PLoS Pathog. 2020 Mar 5;16(3):e1008296. doi: 10.1371/journal.ppat.1008296 (PMC7058292; doi:10.1371/journal.ppat.1008296)
Supplement: Attachment — Submitted filename: Response to Reviewers 112019.docx [file ppat.1008296.s008.docx]

Dear Editor and Reviewers,

We thank the Editor and Reviewers for their many helpful comments. In response to these comments we have added additional data (listed below) and have attempted to communicate more clearly. We recognize the limitations of our work, in that it is generated in an animal model. Much of what we know about the details of HSV pathogenesis has been learned in animal models, however. What we are measuring is not readily examined directly in the human. We anticipate that our findings will lead to new questions about the resolution of HSV reactivation which can be considered when contemplating the pathogenesis of HSV in humans.

Summary of data additions.

**Fig 5E.** We added an additional photomicrograph panel of Iba1 clusters as requested.

**S Fig 2.** We added a figure showing the inverse relationship between intact and fragmented viral protein expressing neurons.

**S Fig 3**. Additional models. We appreciated reviewer 1’s concern that our findings might be unique to the C57BL/6 mouse. We extended our study to include a second mouse strain and also an additional reactivation trigger. We found that fragmentation of viral protein positive neurons occurred in these additional reactivation models.

**S Fig 6.** We added an in vivo intracellular cytokine assay evaluating function of anti-CD4/CD8 treated vs isotype control treated spleen and TG. This assay revealed the anti-CD4/CD8 treatment was associated with loss of IFNγ production in response to viral antigen.

**S Fig 7.** We found that blocking viral DNA replication prevented fragmentation within the 46 hr time frame, revealing that viral protein expression alone was not sufficient to drive neuronal fragmentation in the latently infected TG environment.

Below is our point by point response

Reviewer #1: 1. The main objective of this manuscript is to determine the fate of the neurons in which HSV-1 reactivated. This is a very difficult task due to the low number of neurons in which reactivation is detected. This is not a criticism to the authors, it is more a description of the challenge faced by them. The authors find both neurons that have a normal morphology and others that look “fragmented” upon reactivation.

Although in lines 452-454 the authors suggest that in some neurons the viral cycle will progress and apoptosis will be inhibited, in other sections of the paper such as lines 463-465 it seems that the authors promote the idea that fragmentation and apoptosis of the reactivated neuron is the main mechanism to control reactivation. Even the title of the manuscript suggests this. However, I do not think that this can be concluded from the current data.

We appreciate the reviewer’s comments and have tried to make our ideas and supporting arguments clearer throughout the text. What we are conveying with the title is that at the end of the reactivation event, the neuron, having supported a productive viral lytic cycle, undergoes apoptosis. We have determined that apoptosis is one post-reactivation resolution event, recognizing that we cannot rule out that there are others and we have added this to the manuscript. We are not proposing that apoptosis is controlling the exit from latency (ie, entry into reactivation), but rather characterizes how the reactivation event ends (resolution). In other words, the neuron having supported the full viral lytic cycle, does not survive. In some ways this is not surprising given all we know about HSV lytic infection. However, as we state in our introduction, there are differences in opinion about this. We are unaware of any other study that has directly addressed this question in vivo.

In lines 452-454 we were trying to convey that infectious virus is produced during reactivation, so apoptosis must be initially inhibited while the replication cycle progresses to the point of producing infectious virus. Apoptosis is actively blocked by viral mechanisms during the lytic cycle which would be expected to also function during the lytic cycle associated with a reactivation event. Failure to block apoptosis during the lytic cycle severely impairs infectious virus production. However, we propose that apoptosis does occur subsequent to the production of infectious virus. We propose that this occurs in most (if not all) neurons having undergone reactivation (S Fig. 2). We have added new data supporting this finding (S Fig. 7). Here we show that blocking viral replication with ACV prevents fragmentation during the 46 hr time frame. We have edited this discussion point to increase the clarity of the Discussion.

Similarly, based on data shown in Figure 2, the authors conclude that there is a transition from non-fragmented to fragmented neurons because the percentage of the latter increases with time. However, the total number of fragmented neurons does not increase as one would expect if most non-fragmented neurons become apoptotic.

This would only be true if fragmented neurons are maintained in that state over time. However, clearance of apoptotic bodies occurs rapidly. We have added additional arguments that we hope will make our thinking apparent (218-238) and S Fig 2. The nervous system has an established mechanism of removing apoptotic neurons, critical during development and following pathologic insult.

Since the authors also found the presence of nodules and suggest that these appear in places in which apoptotic neurons were located one would also expect that the number of nodules increases with time reaching a level similar to that of reactivated neurons. The authors show that the number of nodules increases with time (Table 3) but is difficult to compare these numbers with the numbers of reactivated neurons shown in Figure 2 since there is no information on the number of ganglia used for this figure. However, the low number of nodules shown in Table 3 suggests that many of the reactivated neurons did not become nodules. This would suggest that the fate of many reactivated neurons is not to undergo apoptosis and that reactivation is controlled by an alternative mechanism.

We thank the reviewer for bringing this to our attention. The way the data was presented in Table 3, as an average/TG, was misleading and we have removed this table. The number of nodules varied between TG, similar to the number of reactivating neurons shown in Figure 2, however some TG lacked large nodules observable by cresyl violet counterstain alone. The enumeration of the total number of dense cellular clusters (nodules) is inherently difficult on sectioned TG, requiring careful examination of serial sections and judgement with respect to 3 dimensional boundaries of any given cluster. Evaluation of the number of Iba1 clusters using the whole TG approach would allow more accurate quantitative assessment. We have added photomicrographs of how these clusters appear (Fig.5E), but enumeration at every time point would require many more mice. Our more refined analysis of HSV protein positive neurons and presence of cellular cuff provides the correlation.

Figure legends have been updated to provide additional details on the number of samples examined and methods used for data generation.

The authors base their conclusions on a systematic analysis to identify neurons positive to HSV-1 antigen. However, it is possible that HSV-1 reactivated in more neurons without generating enough viral antigens to be detected by antibody staining.

This is an interesting idea, but we suggest an unlikely probability. We do not know of an example in the literature of a cell actively replicating HSV in which the many viral proteins required to carry out this process are below the threshold of detection. It should be noted that the complexity of the antibody “probe” we utilize makes this a highly sensitive approach (multiple epitopes to >14 viral proteins). In the 30 years that we have examined cells, tissues, neurons, etc supporting the viral lytic cycle, we have not a single example where a cell was identified producing infectious virus in the absence of the viral proteins. Even in the case of an abortive infection in which viral DNA replication is inhibited, readily detectable viral proteins are expressed.

The use of hybridization techniques to identify HSV-1 genome and transcripts will detect such neurons allowing the quantification of the percentage of reactivating and apoptotic neurons. This will shed some light on how common the described phenotype is.

Detection of the viral genome and viral RNA does not demonstrate reactivation in the absence of demonstrating progression to protein production and detection of infectious virus. It may reveal, as we already know, that transcripts can be generated from the latent genome in the absence of reactivation. Although of interest, this represents an ambitious study on its own and beyond the scope of the study reported.

2. As indicated in lines 254-257, several cell types are involved in removing cellular debris in peripheral ganglia. However, the authors focus on microglia, a cell type more commonly found in the CNS than in peripheral ganglia (although they can also enter the PNS, i.e., Smith et al., 2019, PLoS Biology and other reports). It may help the reader who considers them as CNS-restricted cells if this is explained in the manuscript.

We are thankful to the reviewer for correcting our misuse of “microglia” to describe the Iba1 expressing tissue resident macrophages. We did not invent the usage of “microglia” for tissue resident macrophages in the PNS, however, we agree it is not correct and have changed this throughout the manuscript. We also agree with the reviewer that it is unlikely that the Iba1 expressing cells have migrated in from the CNS, although this is a possibility which we have added to the Discussion. Iba1^+^ cells are resident in peripheral ganglia and readily detectable in uninfected trigeminal ganglia (Figure 5). We acknowledge that we do not know the origin of cells surrounding reactivating neurons within the TG (lines 565-571)

A possible alternative to the authors’ conclusions is that the Iba1+ cells are not microglia but infiltrating macrophages since they also express this protein (Ohsawa et al., 2004 J Neurochemistry Feb;88(4):844-56; Sasaki et al., Biochem Biophys Res Commun. 2001 Aug 17;286(2):292-7, among others). P2Y12 is expressed in microglia and not macrophages and is currently used as a marker to distinguish between these two cell types. As reported, P2Y12 appears to mark homeostatic CNS microglia Moreover, its expression is increased upon microglia activation. The authors should use this marker to clearly demonstrate that microglia and no macrophages surround the reactivating neurons. Are there other cells, not Iba1+, surrounding the neurons during reactivation? If so, which ones?

We agree that infiltration of macrophages is a possibility and now state this. We are further phenotyping these Iba1+ cells. However, although not included in this manuscript, it is interesting that in RNAseq experiments, we do find TMEM119 and P2Y12 transcripts in TG during latency/reactivation. The expression of P2Y12 has been broadened to include macrophages and other immune cells. (Kloss et al. Cell Death and Disease (2019) 10:760 <https://doi.org/10.1038/s41419-019-2010-6> among others)

Cells surrounding the neuron during reactivation appear to represent a heterogeneous and dynamic population. We cite Shimeld et al. who identified CD4^+^ and CD8^+^ T cells, B cells, and CD11b^+^ and F4/80^+^ macrophages in cellular infiltrate. We acknowledge that the infiltrate surrounding the reactivating neuron is heterogeneous, but Iba1 expressing cells are dominant. A careful characterization of these cells is extremely difficult due to the rarity of the reactivation event and the static nature of the cuff as observed at different time points post-reactivation stimulus. Flow cytometry would certainly allow characterization of cells resident to the ganglia, but this approach excludes localization of the specific infiltrate associated with the reactivating neuron. The location of the cells in the tissue is central to conclusions drawn in this study. We have reported what we have observed which is a large number of 1ba1 expressing cells surrounding neurons as reactivation in the neuron progressed (with increasing time post stress).

One interesting question arising from this work is whether the Iba1+ cells are there to clear the apoptotic neuron or whether they are involved in control of reactivation or in the induction of apoptosis, since non-fragmented neurons are also surrounded by these cells, although to a lesser extent (Figure 4 E, F). Also, in Figure 5 the Iba1+ cells have a different morphology in the mock and latently infected ganglia. In certain areas of the latent ganglion there is more Iba1 expression and the Iba1+ cells have a rounded morphology indicative of activation, whereas in others they look as non-activated cells. A quantification of both cellular phenotypes would facilitate asserting the relevance of these observations.

The reviewer notes the morphological differences that we pointed out in Iba1 expressing cells between mock and latently infected ganglia. This picture is quite complex, including regions in latently infected ganglia that do not appear noticeably different than mock infected, as well as regional variations in the phenotypes of apparently activated cells. We are reporting our observations here and plan to follow up by asking how this picture changes when viral reactivation is blocked at distinct stages. Under these conditions, quantification of these morphologically distinct phenotypes will be of great interest and we plan to do this. The focus of this current study is on the neuronal outcome following a reactivation event. We are pleased to have identified Iba1 expressing cells as a prominent feature of the reactivating ganglion landscape.

According to Figure 5B, the Iba1+ cells also surround the neurons before reactivation and cover nodules where presumably a neuron died during lytic infection. It would be helpful to detect the viral genome by FISH to determine whether there are activated Iba1+ cells already surrounding the latently infected neurons. Also, detection of HSV transcripts by RNA-scope or similar technique will inform whether reactivation already started in some of these neurons but did not progress to production of protein and infectious viral particles. This would indicate whether the Iba1+ cells participate in initial control of reactivation.

These are interesting questions but actually represent another very ambitious set of experiments which, in the end, would be difficult to interpret without a deeper experimental context. Currently both viral DNA and transcripts would need to be performed on sectioned TG, requiring serial sectioning and analysis on most, if not all of the sections. Presumably we would then attempt to localize individual Iba1 cells and correlate their presence with viral DNA and/or transcripts. The significance of a correlation however would be unclear. We already know that Iba1 cells are present surrounding neurons in uninfected ganglia. Further, the large clusters of Iba1 cells are very rare, much rarer than the number of neurons containing the latent viral genome. What we are proposing is that these Iba1+ cells gather around the dying neuron based on release of signals from the neuron.

Once the authors convincingly prove that the cells are microglia or macrophages it will be very interesting to determine their role in neuronal fate upon HSV-1 reactivation by depleting them prior to reactivation. There are protocols available in the literature to deplete either cell population in the mouse.

These types of experiments are on our event horizon but beyond the scope of this current study. Refined approaches to conditionally deplete tissue specific macrophages have been reported (Methods Mol Biol. 2018 ; 1784: 243–258. doi:10.1007/978-1-4939-7837-3_22). As the reviewer points out, further characterization of these Iba1+ cells to identify a potentially unique driver for specific ablation is needed. While these types of experiments are not likely to be straightforward, for example, conditional deletions show incomplete efficacy (Bruttger et al. 2015, *Cell*), these types of studies are certainly an important brick in the path forward.

3. Previous studies did not show efficient reactivation in C57BL/6 mice, contrary to the findings in this report. The authors discuss nicely the possible reasons for these discrepancies. Here, the analysis of protein expression in neurons and the presence of infectious virus in TG demonstrate reactivation. But there is no data showing the presence of HSV-1 in the eye or tear film upon reactivation.

Infectious virus at the body surface represents recurrence subsequent to reactivation. We tested for shedding of virus in tear films, but found it only rarely (Results; lines 420-421). It is possible that using more sensitive methods (PCR for viral DNA) would have revealed viral amplification. Our focus in this study was on the events in the ganglion.

It is possible that C57BL/6 mice are particularly efficient in controlling HSV-1 reactivation and that a different phenotype may be observed in other mouse models in which reactivation is more efficient. Although I am aware of the difficulty of setting up a similar model with a new mouse strain, performing such experiments will allow determining whether the Observations reported here are mouse-strain specific or a general phenomenon. This will also provide information of the mechanism(s) leading to control of reactivation.

This is a good point. We tested this idea using Swiss Webster mice and additionally asked whether the fragmentation was unique to hyperthermic stress induced reactivation. We found that fragmentation was not unique to either C57BL/6 or hyperthermic stress (S Fig.6). Iba1 expressing cells were also prominent. These findings support the hypothesis that our findings in C57BL/6 mice represent a general mechanism for the resolution of reactivation in the neuron in vivo.

Reviewer #2: 1. Overall there were relatively few HSV IHC (+) cell outlines in the entire study, emphasizing careful microscopy. Were the microscopists evaluating from intact vs fragmented HSV protein (+) cell outlines (Fig. 2) blinded as to time point or viral strain? Similarly for Fig. 1C,D were the microscopists blinded for time point? The same blinding query extends to additional data sets and observations.:

We appreciate this reviewers thoughtful concerns. We do recognize the importance of training and experience when interpreting data derived from tissues at the microscopic level. First, it is worth pointing out that a trained pathologist with 30 years of experience is involved in reading out the slides. We routinely employ microscopist blinding and have updated Methods to include this. This study is possible because we are utilizing a whole tissue approach that we refined for the purpose of detecting the rare reactivation event in the ganglia. Viewing the entire ganglion allows us to quantify these rare events with a level of accuracy that would not otherwise be possible, for example on sectioned material. While the numbers of HSV IHC (+) cells are low, the technique for detection is extremely robust. The counts reflect the biology of viral reactivation and are consistent between our whole tissue detection approach and serial sectioning entire ganglia. Every experiment was repeated a minimum of 2-3 times with respect to time point and viral strain with highly reproducible numbers.

2. I am concerned about the interpretation of the data about the temporal progression of inflammatory cuffs surrounding HSV antigen (+) zones over time discussed in lines 291-327. The authors note that at their early time point (20 hours after hyperthermia) there is a mix of cuffed and non-cuffed neuron-like cells, but that by later time points (48 hours) all of the HSV IHC (+) areas (interpreted as neurons) have an inflammatory cuff. Of concern, the absolute number of HSV IHC (+) areas at the late time points is very small, such that the ratio of cuffed to non-cuffed HSV IHC (+) areas is unlikely to be measured with much precision.

If by precision, the reviewer means how close repeated measures are to each other (ie in a different group of animals), the limits of precision would be related to variability in the numerous biological events that occur upstream of the individual reactivation event. We control this by standardizing as much as possible, including uniform infection strategies and uniform application of reactivation stress. We can more readily address accuracy, or does our data reflect what is actually present in the ganglia at 48 hrs post-stress and further, that this reflects the biology? We have included in greater detail how we track a single positive neuron (detected and mapped spatially in the TG using the whole ganglion approach which is extremely robust) through the post-hoc analysis of the infiltrate on the serial sectioned TG. Of the reactivating neurons examined in this way, the cellular context was mapped onto 6/9 of the reactivating neurons detected first in the whole ganglia at 48 h phs.

Between McKrae and 17syn+, there were only 6 HSV IHC (+) areas observed at 48 hours (Tables 1 and 2). Lines 437-438 in the Discussion also concern the proportion of fragmented neurons and this again is based on a very limited number of observations. It is not clear if the nodules discussed in lines 328-335 are really areas of inflammation localized to (now cleared) reactivated neurons: were these nodules also HSV IHC (+),or just clumps of Ib1a1+ cells? ?

While we agree that the number of observations is low, this arises out of the biology of viral reactivation. The reason why we do not know about these basic events is the difficulty in observing them. Even if we double the number of animals in the McKrae group at 48 h phs, we will likely increase the number of observations by 2 fold, yielding 4 HSV IHC (+) neurons. It would be impossible to definitively say that the Iba1 expressing dense cell aggregates (nodules) were previously HSV IHC+ without a time lapse approach in the same host. However, Figure 4 and 5 show HSV IHC+ neurons and cell aggregates that surround HSV IHC+ fragmented neurons. We propose that nodules are related to the resolution of reactivation and not just clumps of Iba1+ cells because these cell aggregates are not detected in uninfected ganglia, mock-infected ganglia, nor uninfected ganglia exposed to hyperthermic stress. The organization of cells within a tissue is critical to biologic function making it highly unlikely that just clumps of Iba1 expressing cells are an irrelevant observation. However, we recognize the limitations and have stated in the text de-emphasized this.

Can we see an example in a micrograph? An average of one such nodule was seen per TG (Table 3) and the legend line 919 indicates that these were “likely” sites of previous neurons. Given the small numbers of these structures and the incomplete documentation that these nodules are sites of cleared neurons, I think these findings should be de emphasized.

We have removed table 3 and de-emphasized these results. We use the term ‘likely’ because as the reviewer points out, how would we definitively demonstrate that these clusters were previously neurons? The nodules are not observed in uninfected TG pre- or post-stress and many other histological studies have identified similar aggregates associated with ganglionic pathologies (Discussion lines 543-550). We appreciate the difficulty in drawing conclusions from rare events, but the data we are presenting is robust. 5/5 events is a 100% outcome, the probability that the next 5 events will be 0% is very low.

3. One of the key conclusions of the paper is that T cells are not involved in the morphologic disruption of HSV IHC (+) areas that probably represented dying reactivated neurons. This is a potentially paradigm-breaking finding in this field, as both prior human and mouse data indicate that HSV-specific T cells form halos around latently infected neurons, and depletion of T cells in various reactivation models leads to increased HSV replication. It would help to build the case for this statement by staining the cell halos around HSV IHC (+) cell areas (as in Fig. 4 F) for T cell markers, in addition to the microglial marker. The simple presence of T cells could not imply a causal role in viral shutdown, but their convincing absence would build the case.

We apologize if T cells not playing a role in neuronal destruction following HSV reactivation was taken as a key conclusion of the paper. Our data does not allow this conclusion to be drawn. We found no evidence for antigen specific T cell function in the resolution of the reactivation event. This is based on the finding that loss of CD4 and CD8 coreceptors did not alter reactivation outcome. We have now shown that fragmentation does not occur within the 46 hr time frame when viral DNA replication is inhibited, despite viral protein expression. Our data does not exclude other T cell functions (Discussion lines 586-595). We have expanded the Discussion to further emphasize potential non-antigen specific contributions of T cells and the potential role of CD4negCD8neg T cells in an attempt to clarify our conclusions. While we were not successful in finding a compatible antibody for marking T cells by IHC in our system, the flow cytometry data would suggest that indeed some T cells are still present in the ganglia following treatment with depleting antibodies. It is important to also consider that previous reactivation models demonstrating an increase in viral replication following T cell depletion were performed ex vivo, a setting in which reactivation is amplified rather than resolved over time.

4. The main technology used was administration of mAbs against CD4, CD8 alpha, and CD8 beta. In preliminary experiments (Sup Fig 2), administration of anti-CD4 and anti-CD8 prior to acute infection leads to increased recovery of HSV at day 7 from eye and brain after eye inoculation. The authors then shift to administering the mAbs 3 days and 1 hour prior to hyperthermic stress in latently infected mice. Fig 7 shows that cells that are CD3(+) TCRbeta positive are still present in spleen and TG at 48 hours after the hyperthermic stress. Thus, the T cells are not depleted: at least some cells are still present, but the CD4 and CD8 epitopes are interpreted as masked by the overwhelming amounts of mAb administered previously to the mice. What we don’t know about in enough detail is what these residual cells are: are they CD4negCD8neg T-like cells (as in some gamma delta T cells or NKT cells) or are they traditional CD4+CD8- or CD4-CD8+ T cells with masked surface CD4/CD8 co-receptors as hypothesized by the authors. We also don’t know if they are functionally blocked by the mAbs.
The human literature contains well described CD8 co-receptor independent T cell clonotypes: for these T cells, the TCR itself can bind strongly to peptide-MHC, and CD8 co-receptor binding to MHC class I is not required for T cell signaling and function. Thus, the absence of stain-accessible CD8 does not mean that residual traditional CD4-CD8+ T cells, if present, are non-functional.

The reviewer is correct in that we do not know what the residual CD3+TCRb+ cells are in the TG. We do not claim that our findings allow us to rule out a role for T cells in general, but that our findings do not support a role for classic CD8+ or CD4+ co-receptor dependent functions which are the ones that have been the focus of most reactivation studies. We have further expanded the Discussion to emphasize the potential role of co-receptor independent T cell functions. We have extended our functional analysis using an in vivo intracellular cytokine assay, to test the effect of the anti-CD4/CD8 antibody treatment on INFγ production (S Fig 6).

We asked whether abortive reactivation would trigger fragmentation in the same time frame as complete reactivation. We have shown previously that treatment with acyclovir to inhibit viral DNA replication during reactivation in vivo prevents infectious virus production but does not prevent viral protein expression. This would reveal whether fragmentation is triggered by viral protein expression per se (ie via immune signaling), or requires downstream events, in this case changes in neuronal homeostasis linked to viral DNA replication. Because gB is expressed early and independent of DNA replication, this major immunodominant peptide would be generated and expected to stimulate CD8+ T cells. However, fragmentation was not observed when viral DNA replication was inhibited. (S Fig 7)

The authors try to address this issue by incubating immune splenocytes with HSV-charged DC as APC in the presence or absence of mAbs (Fig. 7). In these expts, the mAbs are added in vitro; it would have been better to use responder splenocytes from animals that had previously been given mAbs in vivo. They use inactivated HSV to charge the DC, and as expected, they see relatively little CD8 reactivity (little decrement on adding blocking only anti-CD8). DC cross-presentation of inactivated antigen can occur but is typically very challenging to detect. In the data shown, the addition of anti CD8 alone leads to only a partial decrement in T cell activation, while anti-CD4 leads to better blockade that is not increased by adding anti CD8 to the anti CD4.
Thus, we are not really presented data from a system in which anti-CD8 could even potentially block HSV-specific CD8 T cell responses. It would be more convincing to isolate tetramer (+) CD8 T cells specific for the well-known HSV-1 gB immunodominant CD8 epitope and test if the anti-CD8 mabs would block their activation in response to peptide-pulsed or HSV-infected APC, which are more traditional APC for CD8 T cells. Regardless, the use of high doses of candidate blocking mAb in vitro is less convincing than testing responder cells recovered from mAb-treated mice. The finding of neuron fragmentation in the mAb-treated animals given hyperthermic stress could be due to residual CD8 T cell activity that was not depleted/blocked.

We appreciate the reviewer’s explanation for the outcome of this in vitro assay and agree that functional characterization of tetramer isolated CD8 T cells would be more compelling than the simple antigen reactivity test on splenocytes performed in our study. However, we were able to show that when the antibodies were bound to the CD4 and CD8 receptors, the antigen mediated activation of CD4 and CD8 T cells was diminished. Although we agree this assay was not optimum, significant reduction in INFg production was observed. We agree that neuronal fragmentation in mAb-treated mice could be due to residual CD8 T cell activity, although signaling through antigen specific recognition of MHC-I seems unlikely given the outcome of the in vivo intracellular cytokine assay (S Fig 6).

5. In human medicine, there is no evidence of neuron drop-out in post-mortem exams of HSV (+) vs. HSV (-) ganglia. Many neurons in TG are DNA (+) or LAT (+) or both even in very elderly people, consistent with long term survival of at least some infected neurons. There is also no clinical evidence of anaesthesia in lip or genital areas supplied by HSV-infected ganglia that might occur if there was significant loss of neurons. The Discussion should acknowledge the differences in pathogenesis and the difficulty of drawing conclusions in humans.

We have now emphasized that our studies are in mice which may or may not parallel events in humans. It is not possible to know the frequency of reactivation in any individual human or the number of reactivation events over their life span. Our previous mouse work (Thompson and Sawtell 2011, *Journal of* *Neurovirology*) actually demonstrates that repeated reactivation events do not significantly perturb the latent pool. This is because an average of 2-3 neurons reactivate during each event while thousands of neurons remain latently infected. How many of these neurons would need to be lost before aneasthesia at the surface would be anticipated? And since loss occurs over time rather than all at once, it is likely other neurons compensate for individual losses through neurite outgrowth. This phenomenon is supported by human studies of corneas from HSV infected patients (Gallar et al. 2010, *Investigative ophthalmology & visual science*; Hamrah et al. 2012, *Opthalmology*). An important consideration is the stimulation by HSV of IL17C which has been shown to stimulate sensory axonal sprouting and proposed to stimulate repair. This would be expected to offset loss of the neuron by stimulating local axonal branching (discussion line 534-541).

6. Neuron dropout/death in the mouse model was associated with a halo of glial-like cells. Since we don’t know if neuron dropout occurs in humans, the significance of this finding to humans is also unknown. Both host species share the finding of halos or rims of HSV-specific T cells (including HSV tetramer (+) CD8 T cells with markers of cytolytic effector function) that surround HSV-infected ganglionic neurons. Thus there is the potential for T cell recognition of HSV proteins (either in reactivating neurons or surrounding satellite glial cells (SGC)), and either cytolytic or non-cytolytic T cell effector functions influencing the reactivating neuron. In vitro, upon explant, neutralization of CD8 or IFN-g leads to increased lytic HSV reactivation of mouse neurons. Ghiasi et al. have also re-addressed this issue with findings that CD8+ DC may account for some anti-CD8 findings, an issue that should be discussed. This issue of whether or not acquired T cells contribute to control of reactivation in vivo is of great translational concern, as therapeutic vaccines for HSV are largely T cell oriented and current thinking is to try to get antigen-specific T cells into ganglia.

There are several issues with the references the reviewer points to, namely the in vitro approaches utilized. Reactivation is not controlled in an explant setting and infectious virus is amplified rather than eliminated. Indeed, neurons are not destroyed in that context and the translatability to our study is not clear. Ghiasi et al. address important questions as to the role of dendritic cells in controlling HSV reactivation, but again the in vitro context makes their study not directly translatable to ours. We agree that in vivo studies on the role of antigen specific T cells during reactivation is of high importance, since as discussed (lines 544-547) neuronal dropout and death has been detected in humans associated with ganglionic pathology.

Reviewer #3: None

**Part III – Minor Issues: Editorial and Data Presentation Modifications**
Please use this section for editorial suggestions as well as relatively minor modifications of existing data that would enhance clarity.


Reviewer #1: 1. Mouse models are essential to understand HSV-1 pathogenesis. At the same time, some conclusions or observations obtained using mice do not apply to humans. In this report reactivation is rapidly controlled and whether infectious virus is present in the eye or tear film after reactivation is not indicated. Do the authors detect infectious virus in the eye upon reactivation?

The reviewer raises an important point regarding translatability. We have included relevant comments in the text. Indeed, reactivation in humans is difficult to measure since infectious virus at the body surface represents recurrence subsequent to reactivation in the ganglia. We tested for infectious shedding of virus in tear films, but did not detect it in this study (Results; lines 402-404). However, this is not inconsistent with the behavior of the virus in humans. Most seropositive individuals do not display recurrent disease or shed infectious virus. This does not mean that these individuals do not undergo reactivation in the ganglion. It is possible that more sensitive methods (PCR for viral DNA) would have detected viral amplification.

The authors indicate in lines 99-100 that “inflammatory foci have been detected in HSV infected human ganglia post-mortem, independent of viral protein expression (11, 12)”. They also mention in lines 474-476 observations supporting death of neurons after HSV-1 reactivation in humans. However, it is also known that HSV-1 reactivates asymptomatically in humans quite frequently (in some humans but a minor portion of the infected humans), and infectious virus is detected in the innervated mucosa. Moreover, HSV-2 reactivation in human sacral DRG is associated with increased neurite outgrowth and not with neuronal death (Peng et al., J Exp Med 2017, doi: 10.1084/jem.20160581). The data presented here provides support for the death of neurons upon reactivation but, to my knowledge, it is not clear at present what the percentage of reactivated neurons that undergo apoptosis upon reactivation in humans is. Is there a “reactivation threshold” that leads to neuronal apoptosis? Or does this also occur during asymptomatic reactivation? Although it is beyond the scope of this work to obtain answers to these questions, it makes sense to take all these previous observations into account and discuss the relevance in humans of the interesting findings reported here.

We agree and have further expanded the Discussion on human translatability. We thank the reviewer for bringing the study by Peng et al. to our attention. We believe it is consistent with the ideas proposed in our study. Neurite outgrowth is a response to damage. We propose that when the reactivating neuron is destroyed, neighboring neurons exhibit neurite outgrowth to compensate for the loss and avoid loss of innervation or anesthesia at the surface. The findings of Peng et al. would suggest that keratinocytes respond to HSV reactivation by producing IL-17c and this promotes neurite outgrowth by other innervating neurons. Indeed, Peng et al. demonstrate that PRE-treatment of neurons with IL-17c reduces apoptosis following HSV-2 infection: again, only in the context of IL-17c addition prior to infection. This is consistent with our data demonstrating the high specificity of a reactivation event, such that reactivation is limited to an individual neuron and does not result in regional destruction of neighboring neurons. It is possible that the protection of neighboring neurons is related to IL-17c signaling following host detection of the reactivation event, but exploration of this possibility is beyond the scope of our current study.

2. Figure 4: The authors show that 3 out of 3 fragmented neurons are positive for caspase 3 whereas non-fragmented ones are negative. Although the data looks convincing it would be even more so if the n is increased. The caspase 3+ neuron is surrounded by Iba1+ cells. Was this observed in the other apoptotic neurons found?

Yes, all caspase 3+ neurons were surrounded by Iba1+ cells. Caspase-3neg neurons that were HSV+ were also surrounded by Iba1+ cells in the inflammatory cuff, although to a lesser extent (Fig 4).

3. In some experiments, such as the ones in Tables 2 and 3, Figures 2 and 4, it is not clear the number of ganglia used. Also, what is the variation between different ganglia?

Thank you for bringing this to our attention. Some of the Figures are related to each other. We have modified the Figure Legends to clarify the number of ganglia used.

4. Lines 432-435. Maybe I do not understand what the authors imply, but I do not agree with the current statement. The number of HSV-1 positive neurons associated with inflammatory cuff does not increase with time post-reactivation. Tables 1 and 2 show more neurons associated with cuff at 20 hours than later. The percentage is higher but this may be due to the fact that the number of events is highly reduced with time.

We apologize for this oversight. The reviewer is correct and we intended to say percentage is higher, this has been modified.

5. Some references are not inserted with endnote or similar software. Instead, the surname of first author and year are included.

We apologize for this oversight and have corrected the references.

Reviewer #2: 1. Was there a sex difference for 17syn+?

Not with respect to virulence. This has been added to Methods.

2. There are some issues with the Fig. 4 legend. I don’t understand which of the 4 X marks in B correspond to which of the 3 X marked neuros in D. While I can intuit what E is, it is not called out in the legend. Similar for F. G and H appear to be lower power shots of a ganglia with an iba1(+) infiltrate around an HSV (+) cell but this is not called out in the legend. It is called out in the text around line 248 but better to also mention in legend.

We thank the reviewer for calling this to our attention and the Legend has been updated.

3. The morphologic findings in Fig. 5E seem to represent a single finding in a single ganglia, and as such I question whether they should be presented as a characteristic of post-hyperthermic stress samples. In addition, the interpretation in the results lines 289-290 is conjecture, with no visualization of egressing virus, so I suggest this either be relegated to Discussion, or better, dropped. Similarly the line 995-996 statement that the cell cluster might be the site of previously infected neuron in a pre-stress latent ganglia is not supported by data and should be dropped.

This is not an isolated observation, although a dramatic example. We do prefer to include it because it emphasizes our unique view of reactivation in the ganglion in vivo. We often see viral protein in axons during a reactivation event. We acknowledge that this conjecture but and state this in the text with references to support the hypothesis. We have removed the statement regarding cell clusters representing previously infected neuron.

4. The key Fig. 7B, C data showing no difference in reactivation parameters in mAB-treated mice is a little hard to interpret. What virus strain was used and what time point was studied? Of note the number of HSV IHC (+) cells was far lower in Fig 7C untreated animals (1-2 per ganglia) than in the Fig. 1 C, D lead-in experiments: why is this?

The viral strain has been added to the figure legend, ganglia were examined 48 h post-hyperthermic stress. The number of HSV IHC+ neurons is actually very similar in 7C (1-2 per ganglia with 25% ganglia positive) as in Fig 1D lead in experiment (1-2 per ganglia with 29% ganglia positive).

5. The p value in line 447 appears to be based on differences in McKrae vs 17syn+ between Tables 1 and 2 at 20 hours. The mention of 24 hours in line 445 is confusing as 24 hours is not mentioned elsewhere. The numbers of fragmented outlines for McKrae at 20 hours (3, table 1) and for 17syn+ (0, table 2) are very low absolute numbers, such that I question use of stats and drawing conclusions on such small numbers of observations.

The mention of 24 hours has been changed to 20 hours. Although the number of positive events (fragmentation) is low, the number of ganglia examined for reactivation is high, such that conclusions drawn are fairly robust and reflective of the biology of reactivation.

6. As a minor point in Sup 1D were brain dissections and titers done on day 10 in the 17syn+ mice?

Infectious virus was not detected in eyes nor TG on day 8 pi nor in brain samples on day 6 and day 8 pi for 17syn+ infected mice. Based on this data, day 10 was not examined for 17syn+ infected mice, since replication was already resolved.

Reviewer #3: 1) The largest issue is that I believe that the manuscript gives the impression that HSV-1 reactivation itself induces the apoptosis that is being observed and arguing that immune factors (microglia and/or cytokines are not involved). The authors do elude to this possibility in the discussion, but I think it is important enough a point that it be more explicitly stated in the body of the manuscript whether this is discussed as well (for example, lines 264 – 265).

Thank you for raising this point. While we do think macrophages and cytokines play a role in the resolution of reactivation, data supports the hypothesis that it is largely driven by the virus. We have now added an additional experiment where mice are treated with acyclovir prior to reactivation. Although viral protein expression was detected, infectious virus was not produced (replication inhibited) and fragmentation (indicative of apoptosis) was not observed.

2) line 184. It is stated that “less than 2 fold variation” in establishment is detected. This does not seem correct as the variation for each virus seems to be greater than that (lines 186 – 187)?

Thank you, the 2-fold variation has been removed.

3) Related to point 1 above, the way that the title, and conclusions in the abstract and throughout the text of the manuscript read, the authors seem to imply that all reactivating (or antigen-positive) neurons die by apoptosis. While I am convinced from the data presented that a majority of them seem to, the data do not exclude the (likely) possibility that some of them do not. This should be explicitly stated and discussed for completeness).

This possibility has been added to the Discussion.

4) Related to point 4, the sentence on line 528 should be modified to state that “what we can conclude is that at least some neurons supporting HSV-1 reactivation are destroyed by the event”.

We agree, this line is presented as support for a hypothesis rather than a conclusion.

5) Lines 531 – 532. Probably should tine down the statement that suggests CD4+ and CD8+ T cells play no role in maintaining the latent state. As discussed in the paragraph above this statement, they may well play a role in cytokine signaling.

We agree, this has been corrected.
